# Whole-Genome Analysis of *Cryptococcus gattii*, Southeastern United States

**DOI:** 10.3201/eid2206.151455

**Published:** 2016-06

**Authors:** Shawn R. Lockhart, Chandler C. Roe, David M. Engelthaler

**Affiliations:** Centers for Disease Control and Prevention, Atlanta, GA, USA (S.R. Lockhart);; The Translational Genomics Research Institute, Flagstaff, AZ, USA (C.C. Roe, D.M. Engelthaler)

## Abstract

*Cryptococcus gattii* is a recognized pathogenic fungus along the Pacific coast of the United States from California to Washington. Here we report that *C. gattii* may also be endemic to the southeastern United States and has probably been present there longer than in the Pacific Northwest.

The pathogenic fungus *Cryptococcus gattii*, recently designated as a separate species from *Cryptococcus neoformans*, is now recognized as a separate species complex (*1*–*3*). In the United States, *C. gattii* was first identified in culture collections in the late 1960s, when a substantial proportion of *C. neoformans* isolates from California were identified as serotypes B or C (now *C. gattii* serotypes A and B) (*4*,*5*). Since then, *C. gattii* has been identified not only in California but also in the Pacific Northwest (PNW) states of Washington and Oregon, where it is now considered endemic (*6*). Although non–travel-associated cases of infection, typically manifesting as pneumonia or meningitis, are occasionally reported in other areas of the United States (*7*,*8*), *C*. *gattii* has not been considered to be endemic to any other areas of the United States.

## The Study

**Figure 1:**
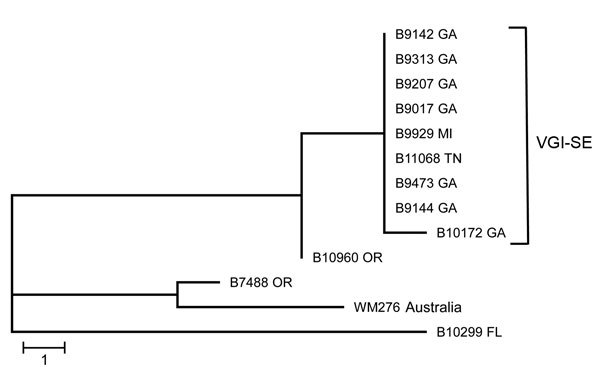
Maximum-parsimony tree of multilocus sequence typing analysis of VGI isolates of *Cryptococcus gattii* from the southeastern United States. In the predominant clade, 1 isolate was from Michigan; all remaining isolates were from the southeastern United States. Nearest neighbor isolates were included for comparison, and an environmental VGI isolate from Australia was used as an outgroup. VGI-SE, VGI southeastern clade. Scale bar indicates 1 single-nucleotide polymorphism.

The Centers for Disease Control and Prevention (CDC; Atlanta, GA, USA) has been performing passive surveillance for *C. gattii* in the United States since 2009. Isolates were collected as part of routine surveillance for *C. gattii* or submitted to the Fungal Reference Laboratory at the CDC for identification. We identified all isolates to the species level as described (*8*) and used the consensus protocol for multilocus sequence typing (MLST) (*9*). In the course of this surveillance, several isolates were received from states in the southeast; genotypic analysis revealed that these isolates consisted of 2 molecular types, VGI and VGIII (*8*). Of >400 isolates received from throughout the United States, 42 were molecular type VGI. MLST analysis of VGI isolates showed a cluster of isolates with a single MLST sequence type originating in the southeastern states (Figure 1). Five isolates from Georgia and 1 each from Florida, Tennessee, and Michigan were indistinguishable by MLST (sequence type 162). A second isolate from Georgia differed from this group by 1 single-nucleotide polymorphism (SNP) in the intergenic spacer gene locus.

**Figure 2:**
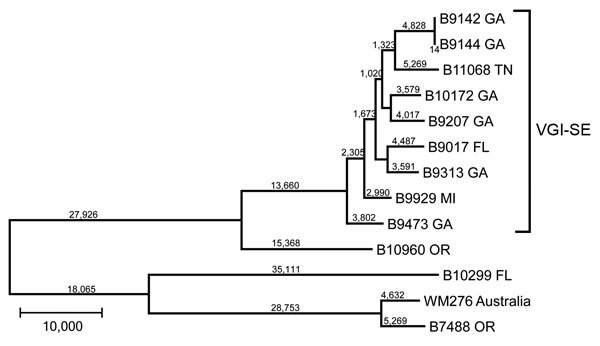
Maximum-parsimony tree of whole-genome sequence data of isolates of *Cryptococcus gattii* from the southeastern United States. All bootstrap values were 100%. Numbers on branches are SNPs. Nearest neighbor isolates were included for comparison, and an environmental VGI isolate from Australia was used as an outgroup. SNP, single-nucleotide polymorphism; VGI-SE, VGI southeastern clade. Scale bar indicates 10,000 SNPs.

Because the population of VGI isolates from the southeastern states (VGI-SE) was only recently identified, it might be a newly emerging population similar to the independent emergences of VGIIa and VGIIc in the PNW (*6*–*8*). To further characterize this cluster, we used whole-genome sequence typing (WGST) of isolates from this cluster, other closely related isolates, and unrelated control isolates (Table 1; Figure 2). We sequenced isolates using Illumina HiSeq or MiSeq technology (Illumina Inc., San Diego, CA, USA), used GATK Unified Genotyper version 2.4 (Broad Institute, Cambridge, MA, USA) for SNP detection, and constructed maximum-parsimony SNP trees using PAUP* (Sinauer Associates, Inc., Sunderland, MA, USA), as previously described (*6*).

**Table 1:** Epidemiologic data for patients in cluster of *Cryptococcus* *gattii*, southeastern United States*

Isolate	State	Year	Age, y	Sex	Disease	Risk factors	Case-patient exposures	Outcome
B11068	TN	2015	62	F	Meningitis	ND	ND	ND
B9929	MI	2012	41	M	Meningitis	ND	ND	ND
B9473	GA	2012	45	M	Meningitis	Sarcoidosis	ND	Survived
B9313	GA	2011	70	M	Meningitis	COPD	Demolished an old shed	Died
B9207	GA	2011	39	M	Meningitis	None	Pressure washed houses	Died
B9144	GA	2011	ND	ND	ND	ND	ND	ND
B9142	GA	2011	ND	ND	ND	ND	ND	ND
B9017	FL	2011	ND	ND	ND	ND	ND	ND
B10172	GA	2013	48	M	Meningitis	HIV	ND	Survived
B10299	FL	2013	61	M	Meningitis	ND	ND	ND
B10960	OR	2014	33	M	Meningitis	None	Previously lived in Guatemala, Texas, and Missouri	Survived
B7488	OR	2009	18	M	Meningitis	HIV	None	Survived
*ND, no data.								

Although the overall topology of the tree we generated remained almost identical to that seen by MLST, we were able to separate each of the isolates within the cluster using WGST (Figure 2). A total of 41,024 SNPs were within the VGI-SE cluster, which had an average branch length of 4,558 SNP differences between any 2 isolates. The isolate from Georgia that differed in MLST by 1 SNP was placed well within the cluster by WGST. Outside the cluster, the nearest neighbor, an isolate collected from a resident of Oregon with a history of living near the southeastern United States in Texas (Table 1), differed by >29,000 SNPs. The other cluster of VGI isolates was separated from VGI-SE by 49,992 SNPs.

Previously, whole-genome sequencing was performed on isolates of *C. gattii* molecular type VGII exclusively from the PNW. Results showed that the VGII isolates consisted of 3 different highly clonal and recently emerged populations with only 107, 132, and 137 SNPs identified within the VGIIa, VGIIb, and VGIIc populations, respectively (*10*) (Table 2). This finding indicated very recent divergence within each of these 3 subtypes (*8*). The average branch length between any 2 isolates was <18 SNPs for the VGIIa and VGIIc populations and only slightly higher for the VGIIb population. On the basis of SNP diversity, the VGI-SE subtype is substantially older than the clonal VGII populations in the PNW and has likely been in the United States for a much longer time.

**Table 2:** Comparison of SNP differences between *Cryptococcus gattii* isolates within the recently emerged VGII clades in the Pacific Northwest and the VGI-SE clade in the southeastern United States*

Genotype	No. isolates	Total no. SNPs	Average no. SNPs between isolates
VGIIa (*9*)	6	107	18
VGIIb (*9*)	4	132	33
VGIIc (*9*)	8	137	17
VGI-SE	9	41,024	4,558

*SNP, single-nucleotide polymorphism.

Two of the isolates from Georgia that we analyzed differed by only 14 SNPs. These isolates, accessed from the same national reference laboratory, were submitted without clinical history; therefore, we cannot discern whether they represent the diversity of 2 isolates from 1 patient over time or whether they are part of a local clonal population.

All of the patients for whom disease manifestation was known had meningitis (Table 1). This finding differs notably from *C. gattii* VGII patients in the PNW, among whom a substantial proportion (59%) had a primary pulmonary infection (*8*). The average age of the patients with isolates in the VGI-SE cluster was 46 (range 18–70) years. For most patients, the source of infection was unknown, although 2 of the patients had been exposed to rotting wooden structures (1 demolished a wooden structure, 1 power-washed old houses).

## Conclusions

Our data indicate that the clonal diversity of the *C. gattii* VGI-SE clade in the southeastern United States is an order of magnitude greater than that seen in isolates from the PNW. Gillece et al. hypothesized that the emergence in the PNW began in the mid-1990s in British Columbia, Canada, and spread to the United States in the early 2000s (*12*). Comparison of the average number of SNPs detected among the 3 VGII clonal groups in the PNW with the average number of SNPs detected between the VGI-SE isolates indicates that the VGI-SE isolates have been in place substantially longer. The limitation to this conclusion is that we do not know the rate of mutation for the VGI or VGII molecular types; however, there is no reason to believe that they would be substantially different. There may also be higher rate of recombination within the VGI-SE clade related to opposite-sex or same-gender sexual recombination. The isolates from this clade for which the mating type is known are all α, but that does not preclude the possibility that some mating type a isolates exist in the environment in the southeastern United States.

The first identified isolate of *C*. *gattii* in the United States was reported from West Virginia in 1924 (*11*), although the isolate was not recognized as *C. gattii* VGI until decades later (*12*). Two other historical reports of *C. gattii* isolates from the southeastern United States exist: the first, from 1968, describes clinical and environmental isolates from Savannah, Georgia; the other, from 1982, describes isolates from Alabama, Tennessee, and Louisiana from patients with no travel history to a *C. gattii*–endemic region (*13*,*14*). Although both of these reports use the previous name for *C. gattii*, *C. neoformans* serotypes B and C, their results clearly indicate that *C. gattii* has existed in the southeastern United States for >40 years, as opposed to the recent emergence in the PNW during the past 2 decades. A more recent report describes a *C. gattii* VGI isolate from an HIV-positive patient in North Carolina (*15*); those authors surmised that the isolate was related to travel to San Francisco, but that assumption may have to be revised. 

No current molecular clock for *C. gattii* indicates the time required to generate the level of diversity seen in the VGI isolates. However, our findings lend credence to the hypothesis that *C. gattii* has circulated in the southeastern United States long enough to be considered endemic.
